# Evaluation and enhancement of suspected opioid overdose definitions in emergency medical services data using machine learning with natural language processing

**DOI:** 10.1371/journal.pone.0347589

**Published:** 2026-04-28

**Authors:** Peter Rock, Svetla Slavova, Sharon L. Walsh, Julia Martin, Daniel R. Harris

**Affiliations:** 1 Institute for Biomedical Informatics, University of Kentucky, Lexington, Kentucky, United States of America; 2 Department of Biostatistics, College of Public Health, University of Kentucky, Lexington, Kentucky, United States of America; 3 Kentucky Injury Prevention and Research Center, University of Kentucky, Lexington, Kentucky, United States of America; 4 Department of Behavioral Science, College of Medicine, University of Kentucky, Lexington, Kentucky, United States of America; 5 Department of Behavioral Science, Center on Drug and Alcohol Research, University of Kentucky, Lexington, Kentucky, United States of America; 6 Department of Emergency Medicine, University of Kentucky, Lexington, Kentucky, United States of America; 7 Institute for Pharmaceutical Outcomes and Policy, Department of Pharmacy Practice and Science, College of Pharmacy, University of Kentucky, Lexington, Kentucky, United States of America; University of Toronto, CANADA

## Abstract

**Background:**

Fatal and non-fatal drug overdoses have evolved into a critical public health crisis, with over a 50% increase in the rate of fatal drug overdose since 2019. Emergency Medical Services (EMS) data has advantages over traditional emergency department data, including timeliness and captured non-transport encounters. However, there is no consensus EMS definition for suspected opioid overdose (SOO), and currently implemented knowledge-based (KB) definition may miss ambiguous cases. Machine learning with natural language processing (ML-NLP) has the potential to enhance SOO identification.

**Methods:**

Secondary data originated from an oversampled dataset of 2,327 weighted encounters from Kentucky State EMS data (2018–2022). EMS experts manually reviewed the records and determined ground truth SOO labels. We examined five commonly accepted KB definitions, ranging from narrow to highly inclusive criteria, spanning from structured-only data to combinations of structured and unstructured data. ML-NLP models were developed considering various EMS data fields and KB indicators. The models and KB definitions were evaluated using sensitivity, specificity, accuracy, precision, and F1-score.

**Results:**

The ML-NLP models outperformed the KB definitions with the structured plus KB model achieving the highest F-score (0.81). Structured-only approaches demonstrated low sensitivity (0.30–0.45). The inclusion of patient care narratives and additional structured fields improved model performance with the ML-NLP models demonstrating high sensitivity (89.1%) and precision (89.0%).

**Conclusion:**

Integrated ML-NLP approaches offer significant improvements in opioid overdose surveillance compared to structured-only, unstructured-only, and KB-only approaches. Future research should explore the generalizability of these models across different populations and geographic areas.

## 1. Introduction

Fatal and non-fatal drug overdoses have evolved into a critical public health crisis, with over a 50% increase in the rate of fatal drug overdose since 2019 [1]. Federal agencies, such as the National Institute of Health (NIH) and the Centers for Disease Control and Prevention (CDC), have invested heavily to address the crisis through initiatives like Helping End Addiction Long-Term (HEAL) [2] and Overdose Data to Action (OD2A) [3], respectively. These efforts emphasized timeliness and completeness of data, crucial for enabling communities, healthcare organizations, and public health agencies to plan and respond to changing dynamics. In surveillance systems, timeliness refers to the rapid availability of data – ideally within hours to days rather than weeks to months and enabling early detection of emerging patterns and clusters. Completeness addresses accurate, comprehensive capture of all cases in an underlying population. Timely and accurate systems are needed to inform issuance of health alerts or public service announcements, targeted distribution of naloxone, strategies to enhance harm reduction, and other rapid response approaches that may mitigate fatal and non-fatal overdoses.

Traditional surveillance of non-fatal opioid overdose relies in part on emergency department (ED) administrative claims from insurance billing data for monitoring and burden reporting which focus on coded ICD-10-CM definitions [4]. While comprehensive and fully integrated in most public health surveillance efforts, these data often suffer from significant delays in data availability and may not capture the full scope of community burden. In the United States syndromic surveillance, a rapid public health surveillance system that typically leverages ED data including chief complaints and discharge diagnosis, is far more timely than claims data and readily integrated into national systems [5]. However, syndromic surveillance likewise does not capture events where patients refuse EMS transport or do not seek hospital care in the event of an overdose.

Emergency medical services (EMS) data are a relatively new data source for public health surveillance, particularly for monitoring and reporting suspected opioid overdoses (SOO) and detecting change in overdose patterns [6–10]. EMS records are typically available within hours to days as compared to months to years for ED administrative claims data. This timeliness difference stems from data flow differences: EMS typically submit electronic reports within hours of a patient encounter. In contrast, administrative claims data involves additional post encounter steps including medical coding, insurance adjudication, and claims submission and aggregation before data availability. Prior research has demonstrated that EMS and ED opioid overdose time series data have significant positive correlation [11]. Uniquely, EMS data captures instances of opioid overdose where the patient refuses transportation to ED, which would not be captured by the ED or ED syndromic surveillance systems [12,13]; while additionally ascertaining address-level residence and incidence location details. The importance of EMS data was clearly demonstrated during recent COVID-19 outbreaks, when patients specifically avoided emergency departments [14,15]. Compared to traditional sources that rely upon ED administrative claims, EMS data has the potential to capture more events and inform public health more rapidly; despite this, consensus adoption of EMS as a surveillance source may be hindered by the numerous competing definitions of SOO.

There is no consensus definition for identification of SOO in EMS data, and using naloxone administration as a sole proxy for SOO has demonstrated limitations [8,16]. More recently, naloxone administration and response are wrapped in as part of expert-developed knowledge-based (KB) definitions, which also include other keywords and elements [6,8,9,17,18] Machine learning with natural language processing (ML-NLP) has been proposed as an alternative to keyword or pattern matching in recent publications [19–21]. These prior ML-NLP implementations demonstrated enhanced capabilities in identifying overdoses but are less informative without comparisons to currently implemented surveillance definitions that may underperform on ambiguous SOO encounters.

This study sought to determine if ML-NLP applied to EMS encounter data could improve identification of SOO by investigating the performance of SOO definitions using data labeled by expert reviewers. Additionally, we examined the performance changes in an ML-NLP classifier as available data elements are enhanced from 1) patient care report narratives (PCRN) only, 2) inclusion of additional fields related to overdose, and 3) direct inclusion of the KB definitions applied to each case. The results aimed to inform the selection of an optimal SOO definition in ambiguous encounters, while evaluating the potential capabilities on ML-NLP, particularly in the context of field availability and currently implemented KB definitions.

## 2. Materials and methods

### 2.1. Data source

This study employed a secondary data analysis approach using existing EMS records (data received on 01/03/2024). Data from EMS encounters in Kentucky from 2018 to 2022 were provided by the Kentucky Board of Emergency Medical Services. These data were originally collected for administrative and operational purposes, not specifically for research. Canceled encounters and interfacility transfers were excluded, as canceled encounters typically occur when EMS units are dispatched but no patient contact occurs and interfacility transfers could result in double-counting. Kentucky EMS data were collected in accordance with the Kentucky administrative regulations [22], following the National EMS Information System (NEMSIS) data standard, v3.4 [23]. EMS records included both structured (coded) and unstructured (free-text) data elements. While NEMSIS data standard encompasses hundreds of data fields, the completeness and quality of individual fields may vary among jurisdictions. With over two hundred licensed agencies conducting more than 550,000 emergency encounters annually, the Kentucky EMS dataset offered a diverse set of EMS run reports. Previous studies have conservatively estimated that SOOs represented approximately 2% of their EMS encounters, corresponding to an estimated 55,000 Kentucky instances within our study period [8,17]. The University of Kentucky Medical Institutional Review Board approved this study with waived informed consent of the fully anonymized dataset (IRB#92233).

### 2.2. Knowledge-based (KB) definitions

Various definitions have been proposed to classify suspected opioid overdose (SOO) in EMS encounters and these definitions varied in their inclusivity, ranging from those relying narrowly on structured fields to more inclusive approaches incorporating unstructured data. This analysis considered five KB definitions with respective structured and unstructured NEMSIS data fields indicated in Table 1. The identification of specific NEMSIS elements needed for each KB definition was informed by the developers of the definitions and their historical data quality and operational experience. The narrowest definition classified when structured medication (eMedications.03) equaled “naloxone” and associated response (eMedications.07) equaled “positive response.” The Office of National Drug Control Policy (ONDCP) Nonfatal Opioid Overdose Tracker [24] expanded the definition with additional structured fields for key matches. Two state-level definitions were considered: Massachusetts Ambulance Trip Report Information System (MATRIS) Opioid Related Incident [25] and Rhode Island Enhanced State Opioid Overdose Surveillance (RI-ESOOS) case definition [26] Both used combinations of key matches in structured and unstructured fields, through expert derived rule-based criteria for inclusion/exclusion. More recently, the Council of State and Territorial Epidemiologists (CSTE) provided a guidance document and definition for SOO in EMS data [27] Although the scope of some definitions may be broader opioid-related encounters, all are considered SOOs in this project. These definitions are subject to updates and change over time; thus, the versions used in this study may differ from newer or subsequent definitions. Notably, the CSTE definition only became available after our sample had been created and was not used as part of the KB set used to generate the sample. However, due to the possible importance and impact of the CSTE definition guidance, it was included in performance analysis and modeling.

**Table 1:** NEMSIS elements used in KB definitions and ML-NLP modeling.

	KB Definitions					ML-NLP		
NEMSIS data fields^a^	Structured Medication Only	ONDCP	RI-ESOOS	CSTE	MATRIS ORI	Narrative-only	Integrated Fields	Full Features
*Structured (coded)*								
eMedications.03 – Medication Administered	X	X	X	X	X		X	X
eMedications.07 – Response to Medication	X	X	X	X	X		X	X
eSituation.11 - Provider’s Primary Impression		X	X	X	X		X	X
eSituation.12 - Provider’s Secondary Impressions		X	X	X	X		X	X
eSituation.09 – Primary Symptom		X		X			X	X
*Unstructured (free-text)*								
eNarrative.01 – Patient Care Report Narrative			X	X	X	X	X	X
eSituation.04 – Complaint			X	X	X		X	X
*Binary flags for matching KB definitions*								X

**Abbreviations**: Knowledge-based (**KB**) definitions; Machine Learning Natural Language Processing (**ML-NLP);** National EMS Information System (**NEMSIS**); Office of National Drug Control Policy (**ONDCP**); Rhode Island Enhanced State Opioid Overdose Surveillance (**RI-ESOOS**); Council of State and Territorial Epidemiologists (**CSTE**); Massachusetts Ambulance Trip Report Information System (**MATRIS**).

^a^NEMSIS Data Dictionary Version 3.4, available from www.nemsis.org

### 2.3. Study sample

Considering the high imbalance in the underlying data due to the low frequency of SOO, we implemented sampling techniques to balance the dataset in preparation for identifying SOO with machine learning. Five filters were hierarchically applied to the EMS data, with cases evaluated sequentially through each filter if not identified as a positive match by previous filters, and each filter was weighted according to the perceived complexity for identifying SOOs (see Table 2). These filters ranged from highly probable (lower weight) encounters directly documenting a SOO to more ambiguous indicators of SOO (higher weight), which may have suggested a SOO occurred. In applied order, this included encounters with the following: naloxone administration with a positive response, unique matches to 1 KB definition, matches for 2 + KB definitions, structured fields indicating possible overdose or drug use, and free-text fields indicating possible overdose or drug use, assuming not classified in prior filters, respectively. Specific terms and matching criteria for the filters were also included in Appendix A. The dataset was further oversampled to ensure the representation of a fifty percent Black population, particularly given Kentucky’s increased overdose rates among Black individuals [28]. This oversampling was designed to enhance the generalizability of our findings to other U.S. jurisdictions with more diverse demographic compositions, as many states and communities have substantially higher proportions of Black residents than Kentucky’s general population. This filtering approach targeted the inclusion of the most ambiguous encounters of SOO to focus on harder to classify encounters. Simple KB matches, disagreements in matches, and common keywords pulled together similar encounters that may have opioid overdose involvement but could have also represented other types of encounters such as denied or confirmed negative overdoses, other drugs such as alcohol or marijuana, suicide attempts, and other altered mental status-type encounters. Once the hierarchical filters were applied, a randomized extract from all possible filtered cases was extracted matching the weights stated in Table 2 and 2,237 were manually reviewed by trained experts.

**Table 2:** Sample selection filter criteria and proportion.

Filter	Description	Target proportion (%)
Structured Medication and Response	Encounters with naloxone administration and positive response.	10%
KB Definition Unique^1^	Encounters matching only one KB definition.	30%
KB Definition Match^1^	Encounters matching two or more KB definitions.	30%
Structured Field Key Matches	Structured fields indicating possible overdose or drug use	15%
Free Text Key Matches	Free-text fields indicating possible overdose or drug use.	15%

^1^Council of State and Territorial Epidemiologists (CSTE) definition for suspected opioid overdose was published after extraction and labeling was completed; knowledge-based (KB) definitions filtering (unique and multiple case match) process excluded CSTE definition.

### 2.4. Ground-truth labeling

For both ML-NLP modeling and KB definition evaluation, “ground-truth” of each encounter in the sample being a SOO was determined by manual expert review. Ground-truth labeling was conducted by a team of two EMS paramedics, an emergency department (ED) medical physician, and a PhD epidemiologist after completing standardized training on labeling criteria. This team used a modified version of previously established criteria [9] (Appendix B). Trained personnel labeled overlapping records (n = 25) and Fleiss’ Kappa was used to measure inter-rater reliability and rater agreement among multiple raters. The full EMS data sample was uploaded to a local HIPPA-compliant REDCap instance, which included comprehensive encounter data based on NEMSIS standards (Appendix C). Each record was assessed and labeled as definite, probable, possible, or not SOO based on the full context of the encounter. Encounters labeled as possible were manually re-evaluated, and those that could not be definitively classified were excluded from the analysis (n = 53, 2.3%). Records identified as definite or probable were considered positive indications of a suspected opioid overdose (SOO).

### 2.5. Machine learning definition

Our ML-NLP strategy used tuned random forests (RF) to examine the overall utility of ML-NLP for classification of SOO in EMS encounters, while also examining the impact of increasing SOO-related data inclusion on prediction performance. As observed in the KB definitions, PCRNs were recognized as a critical element in the EMS record for identifying SOOs. In order to delineate the importance of the PCRN and other SOO-related data documented in the EMS record, we developed three models: 1) **narrative-only model (NO)** – using the PCRN only, 2) **integrated fields model (IF)** – PCRN with common structured and unstructured fields used in KB definitions for identifying SOOs, and 3) **full features model (FF)** – integrated model with additional flags indicating SOO by applying each KB definition to the records. The first two models primarily represented an approach without knowledge base, relying only on the assigned reviewer labels and the inherent data. The FF model combined ML-NLP with the invested effort of KB definitions developed by experts for SOO surveillance.

Unstructured (free-text) fields were preprocessed to remove non-alphanumeric characters and stop words prior to transformation into vectorized representations using term-frequency inverse document frequency (TF-IDF), which assigned greater weight to words that were frequent in a single encounter’s unstructured field but rare across all encounters. Structured data were transformed via one-hot encoding to yield binary values indicating their presence or absence. Both structured and unstructured transformations were joined into a single sparse matrix for modeling. The final matrix was split into 80% training and 20% test datasets. Random forest models were tuned to optimize the F1-score using 5-fold cross-validation to mitigate model overfitting and enhance generalizability. Modeling was conducted using Python and the scikit-learn package.

#### 2.5.1. Final comparison of different overdose definitions.

The final evaluation compared the performance of KB and ML-NLP models. Key metrics (sensitivity, specificity, positive predictive value, and F1-score) were used to assess each model’s performance on the 20% test data. Despite the potential biases introduced by oversampling, these metrics facilitated direct comparisons among definitions, highlighting which is most effective under challenging SOO identification conditions.

## 3. Results

The expert review panel evaluated 2,327 encounters, categorizing N = 690 (30%) as SOO incidents. The inter-rater reliability analysis of the training sample resulted in a Fleiss’ Kappa of 0.72, indicating substantial agreement in scenarios with more than two raters [29]. Table 3 provides a detailed breakdown of the distribution of ground-truth positive encounters as determined by the experts and according to the filters applied or the KB definitions implemented. As expected, the structured medication and response filter had an extremely high ratio of positive encounters. Among the encounters uniquely identified by only one KB definition (n = 667), only a small proportion (16.8%) was determined to be positive. For encounters matching two or more KB definitions (n = 532), 59% were determined to be positive SOOs. The structured and free text filters resulted in almost no encounters being identified as being SOOs, likely due to the hierarchical application of filters where more obvious or true positive encounters would have been already included. Additionally, the reviewed encounters were examined by each KB definition and there were observable differences in the definition-specific proportion of true positive and false positives identified (Table 3). As definitions shifted from narrow to inclusive, the proportion of true positive cases increased from 32.8% to 94.3%; however, there was a concurrent decrease in true negatives identified, from 99% to 66.4% -- indicating a varying performance in overall definition case identification accuracy.

**Table 3:** Suspected opioid overdose classification in sample filters and KB definition.

	Ground-truth Positive SOO (N = 690)	Ground-truth Negative SOO (N = 1,637)
*Sample Filters* *(mutually exclusive)*	N (% of Filter)^2^	N (% of Filter)^2^
Structured Medication and Response (N = 242)	226 (93.4%)	16 (6.6%)
KB Definition Unique^1^(N = 667)	108 (16.2%)	559 (83.8%)
KB Definition Match^1^(N = 532)	338 (63.5%)	194 (36.5%)
Structured Field Key Matches (N = 507)	17 (3.4%)	490 (96.6%)
Free Text Key Matches (N = 379)	1 (0.3%)	378 (99.7%)
*KB definitions* *(not mutually exclusive)*	*N (% of 690)* ^3^	*N (% of 1,637)* ^3^
Structured Medication	226 (32.8%)	1,621 (99.0%)
ONDCP	304 (44.1%)	1,556 (95.1%)
RI-ESOOS	557 (80.7%)	1,413 (86.3%)
CSTE	572 (82.9%)	1,201 (73.3%)
MATRIS ORI	651 (94.3%)	1,087 (66.4%)

**Abbreviations**: Suspected Opioid Overdose (**SOO**); Knowledge-based (**KB**) definitions; National EMS Information System (**NEMSIS**); Office of National Drug Control Policy (**ONDCP**); Rhode Island Enhanced State Opioid Overdose Surveillance (**RI-ESOOS**); Council of State and Territorial Epidemiologists (**CSTE**); Massachusetts Ambulance Trip Report Information System (**MATRIS**).

^1^CSTE definition for suspected opioid overdose was introduced after extraction and labeling was complete, unique and multiple matching involving CSTE definition is excluded.

^2^Sample filter SOO classification proportion presented as row-wise results, for relative comparisons to other filters.

^3^KB definition proportion presented with respect to total ground-truth positive and negative in dataset, for relative comparisons to other KB definition.

The primary analysis of the extracted and labeled dataset revealed varied performance across both KB definitions and ML-NLP models on the 20% test dataset (Table 4), composed of 466 encounters with 136 ground-truth positive SOO encounters. As expected, sensitivity improved as definitions transitioned from more limited (structured-field only type) to more inclusive approaches (e.g., MATRIS); however, this corresponded with an increase in false positives and subsequent decrease in definition-specific positive predictive value (PPV). In general, the inverse relationship between sensitivity and PPV was reflective of a conservative definition bias, where fewer encounters were identified but those cases have a high probability of being true positives. This could be observed in the structured medication and ONDCP definitions which had low (0.3, 0.45) sensitivity with high PPV (0.82, 0.76, respectively). The RI-ESOOS definition emerged as notably balanced, achieving the highest F-score (0.77) among the KB definitions with much higher sensitivity (0.87) and PPV (0.69).

**Table 4:** KB and ML-NLP performance on test dataset.

Definition	Sensitivity/ Recall	Specificity	Accuracy	PPV/ Precision	F1-score
Structured Medication only	0.30	0.97	0.78	0.82	0.44
ONDCP	0.45	0.94	0.80	0.76	0.56
RI-ESOOS	0.87	0.84	0.85	0.69	**0.77**
CSTE	0.90	0.7	0.76	0.55	0.69
MATRIS ORI	0.94	0.64	0.73	0.52	0.67
*ML – NLP Random Forest (RF) models*					
Narrative-only (NO)	0.56	0.96	0.85	0.86	0.68
Integrated Fields (IF)	0.62	0.97	0.87	0.89	0.73
Full Features (FF)	0.79	0.93	0.89	0.82	**0.81**

**Abbreviations**: Knowledge-based (**KB**) definitions; National EMS Information System (**NEMSIS**); Office of National Drug Control Policy (**ONDCP**); Rhode Island Enhanced State Opioid Overdose Surveillance (**RI-ESOOS**); Council of State and Territorial Epidemiologists (**CSTE**); Massachusetts Ambulance Trip Report Information System (**MATRIS**); Positive predictive value (**PPV**).

The ML-NLP models outperformed KB definitions in their ability to identify SOOs. More specifically, the FF RF model outperformed all KB definitions with an F1-score of 0.81. However, there were performance differences in the more basic versions of the RF model. The NO model performed modestly with an F1-score ranking it three out of six compared to the other KB definitions. The lower sensitivity indicated that the lower F1-score might be driven by missed positive encounters, which could be attributed to the absent structured fields. When those additional fields were included (IF model), there was a minor increase in F1-score, placing this model in second place among the KB definitions. The FF model had the best F1-score (0.81) and best accuracy (0.89); the other performance metrics for the FF model demonstrated a more holistically accurate model balancing recall and precision in comparison to KB definitions which experienced observable trade-offs in recall and precision. When directly compared to the top KB definition (RI-ESOOS), the FF RF model had much higher PPV but lower sensitivity, indicating the model was slightly more conservative for positive SOO classification. Looking at overall accuracy, the FF RF model was higher than the RI-ESOOS (89% vs 85%, respectively); indicating it was better at determining true negatives and true positives. The higher specificity in the FF RF model might be important in the context of the nuanced SOO encounters presented in the sampled dataset where identification of negative cases could also be important.

**Fig 1:**
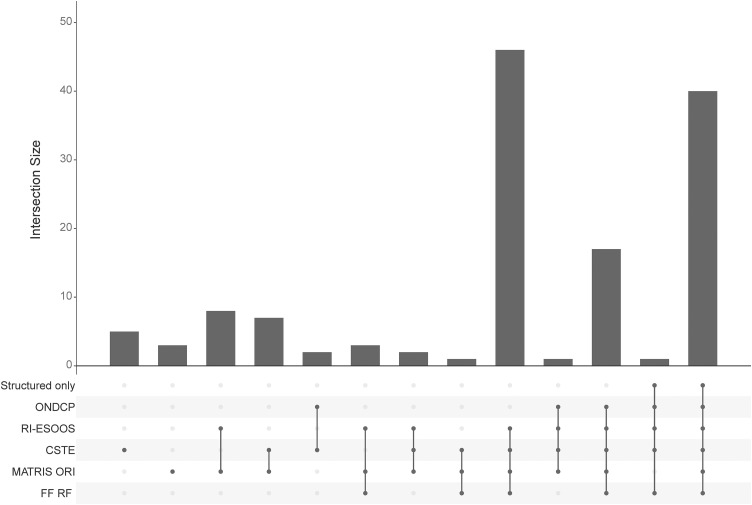
UpSet Graph of KB and ML-NLP Classification Overlap in Test Sample. **Abbreviations**: Knowledge-based (**KB**) definitions; National EMS Information System (**NEMSIS**); Office of National Drug Control Policy (**ONDCP**); Rhode Island Enhanced State Opioid Overdose Surveillance (**RI-ESOOS**); Council of State and Territorial Epidemiologists (**CSTE**); Massachusetts Ambulance Trip Report Information System (**MATRIS**); Full Features Random Forest (**FF RF**) model. UpSet graph develop using *UpSetR* package.

**Fig 2:**
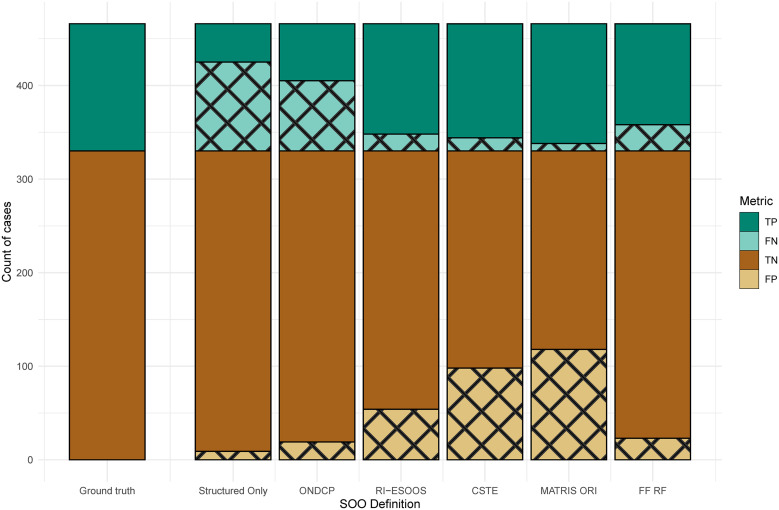
Classification Performance with Respect to Ground Truth in Test Sample. **Abbreviations**: Suspected opioid overdose (**SOO**); Office of National Drug Control Policy (**ONDCP**); Rhode Island Enhanced State Opioid Overdose Surveillance (**RI-ESOOS**); Council of State and Territorial Epidemiologists (**CSTE**); Massachusetts Ambulance Trip Report Information System (**MATRIS**); Full Features Random Forest (**FF RF**) model; True Positive (**TP**), False Negative (**FN**); True Negative (**TN**); False Positive (**FP**).

Fig 1 is an UpSet [30] plot of the ground-truth labeled encounters from the test dataset (n = 136). This graphic visualizes all unique and overlapping matches among the KB definitions and the FF RF model. The bar chart represents the magnitude of the encounters, and the corresponding figure underneath indicates the respective definition intersection, such as the number of encounters matching only RI-ESOOS and MATRIS ORI indicated by two dots with a connected line. The RF model often classified encounters matched by various sets of definitions, serving as a bridging definition. For example, there were a handful of cases where two KB (CSTE/MATRIS, RI-ESOOS/MATRIS) and the RF acted as a common denominator. The RF model overlapped a large host of positive cases, and notably, there was only one encounter where all KB definitions agreed but the RF model missed the classification. Additionally, only CSTE and MATRIS ORI definitions were able to classify encounters uniquely; however, these definitions had high sensitivity and low specificity, and performance was accompanied by many false positives. Definitions-specific performance with respect to true positive (TP), false negative (FN), true negative (TN), and false positive (FP) is indicated in Fig 2, along with the ground truth labeled positive and negative indicated in the first vertical stacked bar. Visually, this figure aids in discerning the increase in proportion of TP (teal) coupled with the increase in FP (light brown with crosshatch). When comparing top performing RI-ESOOS to RF model, the frequency of TP was similar yet the difference in FP demonstrated the RF superior performance.

## 4. Discussion

Our findings highlight the potential and need for ML-NLP tools for classifying EMS SOO encounters, particularly when integrating prior domain knowledge (i.e., KB definitions) as features. The FF ML-NLP model produced the best result, outperforming any of the KB definitions, optimizing a balance of sensitivity and positive predictive value. This highlights the added value of incorporating unstructured data with KB features to enhance classification accuracy. Uniquely, our approach enabled a direct comparison of KB definitions, revealing that RI-ESOOS outperformed other KB definitions in the sample, making it the most reliable standalone definition. To our knowledge, this is the first comprehensive evaluation of KB definitions and ML-NLP models in the context of EMS SOO definition validation. Our results build confidence in the utility of combining KB with ML-NLP model development for SOO classification and underscore the potential of such models for providing accurate SOO estimates in EMS data.

Although our best model achieved an F-score of 0.81, which is lower than other published models [19–21]; this gap may be due to the difficulty-weighted sampling. The approach to focus on challenging cases likely to be misclassified by KB definitions provided a more rigorous test of model performance. As a result, our models demonstrated the strengths of ML-NLP for processing the free-text PCRNs while also leveraging the subject matter expertise embedded in existing KB definitions.

Our findings align with prior surveillance efforts, particularly regarding the role of free-text PCRN data in identification of SOO. Although the NEMSIS standard provides a formal structure to capture encounter data through structured fields, including fields marked as being mandatory, many KB definitions recognize the limitation of relying on such fields which are often incomplete. EMS personnel often document complex scenarios in the PCRNs, resulting in detailed descriptions of the condition and treatment of patients. Our analysis highlights the gaps in sensitivity of structured-only approaches (range 0.30–0.45), demonstrating the limitation of this method. Conversely, sole reliance on the PCRN is insufficient, as demonstrated by the NO RF model’s performance, achieving a sensitivity of only 0.56. The far superior performance of the IF and FF models reinforces the need for combining both structured and unstructured data for more accurate SOO classification.

### 4.1. Implementation considerations

Despite the promising results of the ML-NLP models, technical challenges may still greatly hinder their adoption in public health practice and research utilizing EMS data. Privacy concerns necessitate analysis on premises, which presents a host of software, hardware, and technical expertise challenges in this more closed environment. Additionally, ML-NLP models are typically a black-box where explainability is difficult, which possibly limits trust in model performance – especially if not directly compared to common industry standards. These challenges drive a continued preference for the KB definitions, particularly for individual record level review where a set criteria or flowchart may be more functional. Importantly, this preference is supported by performance: the best-performing KB definition (RI-ESOOS, F1 = 0.77) and the best ML-NLP model (F1 = 0.81) are closely comparable, and for many jurisdictions the modest performance difference may not justify the additional infrastructure investment. In this context, RI-ESOOS represents a practical and sufficient standalone option, particularly where technical resources are limited. However, when analyzing large datasets with ambiguous cases, ML-NLP methods demonstrate superior accuracy. Furthermore, ML-NLP models are often developed on specific jurisdictional data; for example, our model was developed with Kentucky data which can limit generalizability and model governance.

However, there are opportunities to address these challenges narrowly and integrate ML-NLP with KB definition through the lens of the nationally implemented NEMSIS data standard with participating states that submit their data centrally to the national platform. If entities such as ONDCP’s Nonfatal Drug Overdose Surveillance Dashboard were to incorporate PCRN data, they could offer uniform classifications and return record-level results to jurisdictions, supporting the development of more accurate surveillance systems and research datasets.

### 4.2. Limitations

This study has several limitations that may impact generalizability. The analysis was based on Kentucky data, which may not be representative of other jurisdictions. SOO was defined as positive for definitive and probable encounters, excluding a small portion of possible encounters. Although this choice aimed to enhance precision, it may have overlooked potentially relevant cases.

Although reviewers showed moderate agreement in reviewer training set, there is potential for improved consistency by double reviewer labeling of all records with arbitration. Our evaluation did not include more advanced models such as large language models; additional machine learning models were tested with similar performance as the RF, specifically: naïve Bayes, support vector machine, and gradient boosting machines.

We are limited to a snapshot in time when using the KB definitions, which may have been updated since inception of this study. This may not detract from the goal of comparing KB definitions, but it does limit interpretation with respect to their current versions. This study did not consider model performance concerning specific demographics or geographic subgroups, which could reveal important performance differences in identifying SOO in specific subgroups.

### 4.3. Future directions

Future research should focus on external validation of these ML-NLP models across diverse geographic and demographic groups. Systematic evaluation of model performance across demographic subgroups (age, sex, race/ethnicity, urban vs. rural) can ensure accuracy across populations. Similarly, temporal validation studies can evaluate model accuracy with shifting drug supply and overdose patterns changes.

## 5. Conclusion

Our study underscores the need for integrating domain-specific knowledge with advanced ML-NLP techniques to improve the identification and classification of SOO encounters in EMS data, ultimately enhancing public health surveillance. We demonstrate the feasibility of building an accurate model for SOO, while presenting a replicable standards-based approach that can extend to other conditions.

### Declaration of generative AI and AI-assisted technologies in the writing process

During the preparation of this work the author(s) used Claude and ChatGPT as grammar-checking tools in the late stages of drafting the document. After using these tools, the author(s) reviewed and edited the content as needed and take(s) full responsibility for the content of the published article.
